# Cervical dilatation at diagnosis of active phase of labour determines the mode of delivery and peripartum outcomes: a retrospective study in a single tertiary centre in Malaysia

**DOI:** 10.1186/s12884-023-05523-7

**Published:** 2023-04-01

**Authors:** Anizah Aishah Rosli, Azmawati Mohd Nawi, Ixora Kamisan Atan, Aida Mohd Kalok, Shuhaila Ahmad, Nor Azlin Mohamed Ismail, Zaleha Abdullah Mahdy, Rahana Abd Rahman

**Affiliations:** 1 Department of Obstetrics & Gynaecology, Universiti Kebangsaan Malaysia, Jalan Yaacob Latif, Bandar Tun Razak, Cheras 56000 Malaysia; 2 Department of Obstetrics & Gynaecology, Hospital Canselor Tuanku Muhriz, Jalan Yaacob Latif, Bandar Tun Razak, Kuala Lumpur 56000 Malaysia; 3 Department of Statistics, Universiti Kebangsaan Malaysia, Jalan Yaacob Latif, Bandar Tun Razak, Cheras 56000 Malaysia

**Keywords:** 4 cm, 6 cm, Cervical dilatation, Active phase of labour, Caesarean section

## Abstract

**Background:**

There is an increasing trend of Caesarean section rate in Malaysia. Limited evidence demonstrated the benefits of changing the demarcation of the active phase of labour.

**Methods:**

This was a retrospective study of 3980 singletons, term pregnancy, spontaneous labouring women between 2015 and 2019 comparing outcomes between those with cervical dilation of 4 versus 6 cm at diagnosis of the active phase of labour.

**Results:**

A total of 3403 (85.5%) women had cervical dilatation of 4 cm, and 577 (14.5%) at 6 cm upon diagnosis of the active phase of labour. Women in 4 cm group were significantly heavier at delivery (*p* = 0.015) but significantly more multiparous women were in 6 cm group (*p* < 0.001). There were significantly fewer women in the 6 cm group who needed oxytocin infusion (*p* < 0.001) and epidural analgesia (*p* < 0.001) with significantly lower caesarean section rate (*p* < 0.001) done for fetal distress and poor progress (*p* < 0.001 both). The mean duration from diagnosis of the active phase of labour until delivery was significantly shorter in the 6 cm group (*p* < 0.001) with lighter mean birth weight (*p* = 0.019) and fewer neonates with arterial cord pH < 7.20 (*p* = 0.047) requiring neonatal intensive care unit admissions (*p* = 0.01). Multiparity (AOR = 0.488, *p* < 0.001), oxytocin augmentation (AOR = 0.487, *p* < 0.001) and active phase of labour diagnosed at 6 cm (AOR = 0.337, *p* < 0.001) reduced the risk of caesarean delivery. Caesarean delivery increased the risk of neonatal intensive care admission by 27% (AOR = 1.73, *p* < 0.001).

**Conclusions:**

Active phase of labour at 6 cm cervical dilatation is associated with reduced primary caesarean delivery rate, labour intervention, shorter labour duration and fewer neonatal complications.

## Background

Management of labour is challenging and as a result, partogram was created to guide obstetricians towards safe labour management [1]. Various training and labour protocols use Friedman’s original partogram. A normal labour is characterised by the latent phase which has a nearly flat slope and it is not related to the remaining part of labour. Accelerated cervical dilatation follows with major changes seen within 3.5 to 8.5 cm. However, it is important to diagnose arrested labour accurately as it may lead to unnecessary caesarean delivery (CD).

World Health Organization (WHO) had set the optimal rate for CD at 10–15% rate of all births [2]. Over the decades, there is a rising trend of CD rate worldwide, including Malaysia. Karalasingam et al. reported increasing caesarean delivery rate from 21.8 to 25.3% from 2011 to 2015 [3]. The Robson criteria that was used to classify the births showed an increasing trend of CD amongst nulliparous and multiparas women at term in spontaneous labour. Although various attempts were made to reduce the CD rate, there has not been a tremendous reduction seen, most likely due to multiple factors. The decision for CD depends on the practice of different centres and individual obstetricians. This gives rise to a wide variation in the CD rate even for Malaysia [3].

In recent years, researchers had challenged the Friedman’s labour curve and partogram. Advancement in choice of painkillers, characteristics of labouring women and management methods contributed to the need in revising the partogram. In 2002, due to the difference in labour management particularly in the oxytocin use and epidural analgesia Zhang et al. had proposed a gradual transition from latent to active phase of labour. This is in contrast to the Friedman curve. It takes longer for the cervical os to progress from 4 cm to full dilatation of 10 cm. On the other hand, the rate of cervical dilatation doubled after 5 cm [4]. The median duration of labour prior to 6 cm was similar between nullipara and multipara women. Subsequently, multiparas progress faster than the nulliparas [5]. In view of these findings, the objective of this study is to compare the maternal and neonatal outcomes between women diagnosed in active phase of labour at 4 versus 6 cm cervical dilatation.

## Materials and methods

We retrospectively reviewed deliveries from 1^st^ January 2015 until 31^st^ December 2019, in a single tertiary centre, Universiti Kebangsaan Medical Centre (UKMMC). We obtained institutional review board approval from the hospital ethics committee (FF-2021–087). The study population involved all women who had cervical dilatation of 4 or 6 cm. All methods performed were in accordance with the relevant guidelines and regulations. The data was extracted from the labour room central management system. The inclusion criteria were all pregnant women at and above 18 years old, singleton pregnancy with cephalic presentation admitted in spontaneous labour at 37 weeks gestation or more with cervical dilation of 4 and 6 cm with or without intact membranes. The exclusion criteria were medical disorders such as diabetes and hypertension, fetal complications such as small for gestational age, fetal growth restriction, oligohydramnios, polyhydramnios, patients who had labour induction and previous uterine scars. In our centre, women who achieved 4 cm or more would be sent to labour room according to the hospital protocol. Amniotomy is performed by the doctors if the membranes are still intact. This is followed by oxytocin infusion to augment labour when the contractions are not optimised after 2 h.

We analysed information on maternal demographic and clinical characteristics such as maternal age, ethnicity, maternal weight at delivery, parity and gestational age at delivery, intrapartum management details such as use of oxytocin infusion, analgesia, duration of the active phase, mode of delivery and neonatal outcome such as birth weight, Apgar score at 5 min, arterial cord pH at birth and requirement for neonatal intensive care unit (NICU) admission.

### Statistical analysis

Data entry and statistical analysis was performed using the Statistical Package for Social Sciences (SPSS) software (version 25.0). The relationship between studies variables was analysed using appropriate statistical analysis. The mean and standard deviation was used for continuous variables while frequency and percentages for categorical variable. The student’s t-test was used to ascertain the significance of differences between mean values of two continuous variables with normal distribution. Chi-square or Fisher’s Exact test was used to determine the associations between individual categorical independent factors and outcomes. The level *p* < 0.05 was considered as the cut-off value for significance. The relationships between variables and comparability of groups (4 cm vs 6 cm) with the outcome of interest (maternal and fetus) were tested individually using linear regression.

## Results

**Fig. 1:**
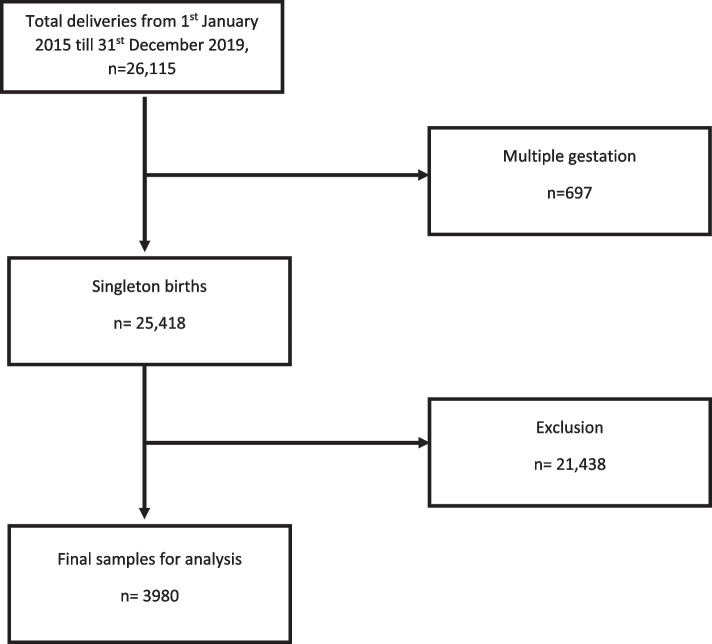
Diagram of sample selection

There was a total of 26,115 deliveries during the study period. Only 3980 fulfilled the inclusion criteria and included in the final analysis as depicted in Fig. 1. Table 1 compares the demographic data and clinical characteristics between women with cervical dilatation of 4 and 6 cm. A total of 3403 (85.5%) had cervical dilatation at 4 cm, and 577 (14.5%) at 6 cm. There were no significant differences in the mean maternal age, mean gestational age at delivery and ethnicity. However, women in the 4 cm group were significantly heavier at delivery (*p* = 0.015) whilst there were significantly more multiparaous women in the 6 cm group (*p* < 0.001).

**Table 1:** Comparison of demographic data and clinical characteristics between women with cervical os dilatation of 4 cm and 6 cm, *n* = 3980

	**Cervical dilatation**		***p*-value**
	**4 cm *n* = 3403**	**6 cm *n* = 577**	
Mean maternal age ± SD, years	29.3 ± 4.4	29.5 ± 4.5	0.343^a^
Group Age (years), n (%)			0.205^b^
18–29	1849 (54.3)	296 (51.3)	
30–39	1503 (44.2)	268 (46.4)	
≥ 40	51 (1.5)	13 (2.3)	
Mean maternal weight at delivery ± SD, kg	69.7 ± 12.3	68.3 ± 12.1	0.015^a^
Mean gestational age at delivery ± SD, weeks	39.2 ± 1.0	39.1 ± 1.0	0.917^a^
Ethnicity, n (%)			0.125^b^
Malay	2568 (75.5)	418 (72.4)	
Non-Malay	835 (24.5)	159 (27.6)	
Chinese	477 (57.1)	93 (58.5)	
Indian	66 (8.0)	6 (3.8)	
Others	292 (34.9)	60 (37.7)	
Parity, n (%)			< 0.001^b^
Nulliparous	1251 (36.8)	170 (29.5)	-
Multiparous	2152 (63.2)	407 (70.5)	

*SD* Standard deviation

^a^Independent t-test

^b^Chi-square test

Table 2 shows the intrapartum management and labour outcomes between the two groups. There were significantly fewer women in the 6 cm cervical dilatation group who needed oxytocin infusion (*p* < 0.001) and epidural analgesia (*p* < 0.001). There were also significantly lower CD rate (*p* < 0.001), CD done for fetal distress and poor progress (*p* < 0.001 both). The mean duration from diagnosis of active phase of labour until delivery was significantly shorter in the 6 cm group (*p* < 0.001) with lighter mean birth weight (*p* = 0.019). Fewer neonates delivered had arterial cord pH of less than 7.20 (*p* = 0.047) or requiring neonatal intensive care unit (NICU) admission (*p* = 0.01). There were no statistical significant differences in meconium-stained liquor, 3rd and 4th degree perineal tear, postpartum haemorrhage, Apgar score at 5 min less than 7 and meconium aspiration syndrome.

**Table 2:** Intrapartum management and labour outcomes between women with cervical os dilatation of 4 cm and 6 cm, *n* = 3980

	**Cervical os dilatation**		***p*-value**
	**4 cm** ***n* = 3403**	**6 cm** ***n* = 577**	
Meconium stained liquor at amniotomy, n (%)	193 (5.7)	28 (4.9)	0.427^b^
Oxytocin augmentation, n (%)	2098 (61.7)	100 (17.3)	< 0.001^b^
Use of epidural analgesia, n (%)	683 (20.1)	23 (4.0)	< 0.001^b^
Mean duration from active phase of labour to delivery ± SD, hours	4.1 ± 2.7	1.6 ± 1.5	< 0.001^a^
Caesarean section, n (%)	490 (14.4)	21 (3.6)	< 0.001^b^
Caesarean section for fetal distress, n (%)	337 (68.8)	14 (66.7)	< 0.001^b^
Caesarean section for poor progress of labour, n (%)	131 (26.7)	4 (19.0)	< 0.001^b^
3^rd^ and 4^th^ degree perineal tear, n (%)	11 (0.4)	3 (0.5)	0.443^c^
Postpartum haemorrhage, n (%)	97 (2.9)	11 (1.9)	0.197^b^
Mean birth weight ± SD, g	3133.3 ± 359.2	3171.3 ± 352.6	0.019^a^
Apgar score at 5 min < 7, n (%)	17 (0.5)	0 (0.0)	0.157^c^
Arterial cord pH ≤ 7.20, n (%)	344 (10.1)	42 (7.3)	0.047^b^
Admission to NICU, n (%)	523 (15.4)	65 (11.3)	0.01^b^
Meconium aspiration syndrome, n (%)	8 (4.1)	0 (0.0)	0.612^c^

*NICU* Neonatal intensive care unit, *SD* Standard deviation

^a^Independent t-test

^b^Chi-square test

^c^Fisher’s exact test

Table 3 shows multivariable analysis to determine the relationship between the mode of delivery by lower segment caesarean section (LSCS) and other confounding factors. An increase in 1 kg of maternal weight at delivery increased 1.0 time risk for delivery via caesarean section. Multiparous women have 51.2% reduction of risk for CD as compared to nulliparous. Oxytocin augmentation reduced 51.3% risk of delivery via caesarean section. Cervical dilatation of 6 cm reduced the risk of CD by 66.3% as compared to 4 cm.

**Table 3:** The associated factors with the mode of delivery (*n* = 3980)

Variable	Multivariate	
	**AOR (95% CI)**	***p*-value**
Mean maternal weight at delivery, kg	1.015 (1.006, 1.024) Increase in 1 kg maternal weight will increased 1.5 times for LSCS	0.001
Parity		< 0.001
Nulliparous	I (Reference)	-
Multiparous	0.488 (0.387, 0.617)	
Oxytocin augmentation		< 0.001
No	1 (Reference)	-
Yes	0.487 (0.350, 0.678)	
Duration from admission to labour room to delivery, hours	1.126 (0.745, 1.702)	0.575
Cervical dilatation at diagnosis of active phase of labour		< 0.001
4 cm	1 (Reference)	-
6 cm	0.337 (0.202, 0.564)	

*AOR* Adjusted odds ratio, *LSCS* Lower segment caesarean section, *SVD* Spontaneous vaginal delivery

Table 4 shows multivariable analysis to determine the relationship between NICU admission and other confounding factors. Delivery via caesarean section increased 27% the risk of NICU admission as compared to vaginal delivery. Increase in 1 g of the neonatal birth weight will increase 1.0 time risk for NICU admission.

**Table 4:** The associated factors with NICU admission (*n* = 3980)

Variable	Multivariate	
	**AOR (95% CI)**	***p*-value**
Mean maternal weight at delivery, kg	0.995 (0.987, 1.003)	0.117
Parity		0.229
Nulliparous	1 (Reference)	
Multiparous	0.882 (0.719, 1.082)	
Oxytocin augmentation		0.415
No	1 (Reference)	
Yes	1.114 (0.859, 1.445)	
Rate of cervical dilation per hour	3.983 (0.594, 26.680)	0.151
Cervical dilatation at diagnosis of active phase of labour		0.054
4 cm	1 (Reference)	
6 cm	0.746 (0.554, 1.005)	
Mode of delivery		< 0.001
Vaginal	1 (Reference)	-
Caesarean section	1.730 (1.358, 2.205)	
Mean birth weight, g	1.000 (0.999, 1.000)	0.005

*NICU* Neonatal intensive care unit, *AOR* Adjusted odds ratio

## Discussion

The CD rate in our centre had increased for the last five years from 33.0% in 2015 to 36.8% in 2019. This rate is higher than the 10–15% rate recommended by World Health Organization (WHO) [2]. Similar trend was also observed in other countries such as Nepal, Vietnam and China [6–8]. The common indications were previous caesarean section, cephalopelvic disproportion and fetal distress [7]. Severe maternal morbidity resulting from CD such as haemorrhage requiring hysterectomy or blood transfusion, anaesthetic complications, venous thromboembolism, major puerperal infection, wound disruption and haematoma was higher in planned caesarean delivery (27.3%) as compared to planned vaginal birth (9.0%) [9]. In addition, the long term impacts are higher risk for placenta previa major and morbidly adherent placenta in subsequent pregnancies [10].

Our study was powered to show a reduction of 10.8% and 7.7% for overall caesarean delivery and caesarean section for poor progress of labour respectively (power > 80%). The change in the demarcation of active phase of labour may be a solution to reduce primary caesarean deliveries for women. Unfortunately, due to the advancement in medicine, caesarean section seemed to be approved as a safe method of delivery. This can be seen amongst the women who delivered in our centre whereby some had requested for ceasarean section during the intrapartum period without a clear indication.

Our study analysed the pregnancy outcomes when the demarcation of active phase of labour is changed from 4 to 6 cm in low risk women who came in spontaneous labour. Active phase of labour demarcated at 6 cm cervical os dilatation seemed to be beneficial with better perinatal outcomes. Zhang et al. discovered that labour takes longer to progress in those with cervical dilatation of 4 and 5 cm at amniotomy as compared to 6 cm. The pace of labour was similar for nulliparas and multiparas before 6 cm cervical dilatation but faster for multiparas after then [5]. This explains our findings of overall CD rate and those done for poor progress was significanty lower in the 6 cm group as compared to 4 cm. The mean duration of active phase of labour was also significantly shorter in women with 6 cm dilatation. Likewise, Thuillier et al. found that the CD rate was lower when the active phase was changed from 4 to 6 cm of cervical dilatation (6.9% vs 9.4%). The CD rate for arrested first stage of labour was also lower by 50%. This reduction was not associated with increase in maternal and neonatal complications such as post-partum haemorrhage, 3^rd^ and 4^th^ degree perineal tear, cord pH < 7.10, poor neonatal Apgar score at 5 min and NICU admission.

We did not investigate whether the progress of labour was influenced by routine amniotomy performed for those with intact membranes when active phase of labour was diagnosed. We are aware that the practice of routine amniotomy is not recommended by WHO and systematic reviews [11, 12]. Routine amniotomy does not reduce the caesarean section rate but decreases labour dystocia [13, 14]. In combination with oxytocin, it is associated with reduction of caesarean section rate [15]. On the other hand, the use of oxytocin is not without risk. It is associated with hyperstimulation and risk of uterine scar dehiscence in women with previous caesarean section [16].

The study was not powered to assess the perinatal safety of changing the demarcation of active phase of labour. However, the trend in the perinatal outcome showed significantly fewer neonates required NICU admission and born with umbilical cord pH lower than 7.20. There were no differences in Apgar score less than 7 and meconium stained liquor leading to meconium aspiration syndrome. These findings concurred with a study by Wilson-Leedy LG et al. [17]. They found no differences in the 5-min Apgar score less than 5 and umbilical cord pH less than 7.

The strength of our study is the large sample size and homogeneity of our population i.e. only low risk women without uterine scar or medical disorders. Hence we are able to capture the reduction in the primary caesarean delivery amongst both nulliparous and multiparous women. However, the limitation of a retrospective study is we are unable to investigate the details of intrapartum management such as duration of the latent phase of labour and duration of oxytocin infusion. There can also be associated bias in relation to selection of patients and missing data. As the study was a retrospective record review data and the practice in our centre of routine amniotomy, there was higher percentage of respondents with 4 cm cervical os dilatation compared to 6 cm. It is an unavoidable scenario. This finding is already controlled in multivariable analysis to justify the conclusion. A larger prospective randomised controlled study is needed to confirm the safety of both mother and neonates with similar number of 4 cm and 6 cm cervical os dilatation for better comparison.

## Conclusion

In conclusion, active phase of labour demarcated at 6 cm cervical dilatation in low risk women with spontaneous labour demonstrated reduced primary caesarean delivery rate, labour intervention, shorter duration of labour, and fewer fetal complications. Change in the demarcation of the active phase of labour is a possible solution to be practised in order to reduce the overall caesarean delivery rate.

## Data Availability

The datasets used and/or analyzed during the current study are available from the corresponding author on reasonable request.

## Abbreviations

CD: Caesarean delivery
NICU: Neonatal intensive care unit
UKMMC: UKM Medical Centre
WHO: World Health Organization
SD: Standard deviation
AOR: Adjusted odds ration
LSCS: Lower segment caesarean section
